# Difficulties in controlling HIV and TB infections in multi-experienced patients

**DOI:** 10.1186/1471-2334-14-S4-P30

**Published:** 2014-05-29

**Authors:** Șerban Benea, Elisabeta Otilia Benea, Mihaela Ionică, Daniela Camburu, Liana Gavriliu, Georgeta Ducu, Alina Cozma

**Affiliations:** 1 National Institute for Infectious Diseases “Prof. Dr. Matei Balş”, Bucharest, Romania; 2 Carol Davila University of Medicine and Pharmacy, Bucharest, Romania

## 

The treatment of extra pulmonary tuberculosis in HIV infected patients experienced to antiretroviral therapy raises major issues of adherence, drug interactions, side effects and limited antiretroviral alternatives.

This is the case of a 24 year old woman from countryside, most probably infected in 1989. She was diagnosed with HIV infection in 2000 after repeated admissions in hospital for pneumonia. She started ART when diagnosed with a favorable evolution: CD4 cell count increased from 200 to 674 cells/cmm, and HIV RNA levels became undetectable. She turned 18 in 2008 and refused to take her treatment for almost two years. She returned to the hospital in April 2010 with a CD4 cell count of 140 cells/cmm. She started again antiretroviral therapy with ABC+3TC+FPV/r.

In April 2013 she is admitted with night sweats, chills and fever. At that time her CD4 cell count was 144 cells/cmm and had 461,000 copies/mL RNA-HIV. Her chest XR showed right pleural effusion, widened mediastinum and the CT revealed infiltrative-like lesion at subcarinal and retrocarinal level. Biopsy was performed and she was diagnosed with ganglionary tuberculosis. She started anti-TB treatment HRZE 7/7 and the ART was changed to ABC+RAL+T20.

The anti-TB regimen is changed after three months to rifampicin and isoniazid 7/7 because she accused arthralgias, tingling of the extremities, blurred vision and decreased visual acuity. She was diagnosed with hyperuricemia (pyrazinamide) and optic and peripheral neuritis (ethambutol).

New visit at the clinic for fatigability, after 7 months of antiretroviral therapy with ABV+RAL+T20. The CD4 cell count was 9 cells/cmm and RNA-HIV was 61,000 copies/mL. She admits taking 1 cp of Raltegravir BID, but denies any other adherence problem. The resistance test showed evidence of resistance to some NRTIs, to all NNRTIs, to RAL and no resistance to PIs. The tropism test showed that maraviroc can be used. We have excluded relapse of tuberculosis and other diseases associated. The ART was changed again to TDF+DRV/r+MRV and the anti-TB treatment is change to isoniazid and moxifloxacin.

Adherence can be an important issue when treating patients for long periods of time. Other important issues are the drug-drug interactions (rifampicin – PIs) and the increased side effects which may lead to loss of good friends (Raltegravir). The poor outcome for this patient may be due to poor HIV and tuberculosis control.

